# On the Bite of a Snake Cured by Volatile Alkali

**Published:** 1804-04-01

**Authors:** David Ramsay

**Affiliations:** Charleston, America


### On the Bite of a Snake cured by Volatile Alkali.

*By* David Ramsay, M. D. *of Charleston, America.*

A Number of extraordinary cures performed within the last twenty years, in the East Indies, on persons bitten by snakes, have been communicated to the public in Jones's Asiatic Researches. These were effected by eau de luce, or by volatile caustic alkali. Similar cures are recorded in Anderson's Recreations, as having taken place in Pondicherry in 1798 and 1799. About the same time my much esteemed friend Mr. Peale, of Philadelphia, added a living rattlesnake to his valuable museum, and invited physicians and others to subject animals to its bite, with a view of determining, by subsequent experiment, the comparative merits of the different remedies commonly recommended for obviating the effects of the bites of venomous animals. The result proved, that the volatile alkali was entitled to a decided preference. Possessed of these facts, I have for some years past embraced every opportunity for ascertaining, by experiment, how far the bites of snakes, or the stings or bites of other venomous animals, might be alleviated by this powerful remedy. A few cases have occurred in my practice, both from the bites of snakes and from the stings of spiders, in each of which the result equalled the recorded beneficial effects of similar applications on the other side of our globe. The last was the case of a negro fellow, by name Stepney, who on the 3d instant was bitten by a rattlesnake at Health Farm, on Charleston Neck. I was not present; but my provisional directions were so punctually carried into effect as to save a valuable life, that in all probability would otherwise have been lost. The experiment was decisive; for though no other application than the volatile alkali was used, the most excruciating agonies of the patient were speedily relieved, and a complete cure obtained in a few days. From full conviction of the efficacy of the remedy, I recommend to planters, and others exposed to the bites of snakes, to have always at hand six or eight ounces of the strongest spirits of hartshorn, well secured; and in case of a person being bitten by a snake, to give him 60 drops thereof in water, every six or eight minutes, till his pains begin to abate, and then to lengthen the interval between the doses in proportion to the abatement of the pain. The wounded part should also be frequently washed with the same medicine. The spirit of hartshorn is particularly designated, because the planters are are acquainted and generally provided with this medicine, and can command it in all seasons and places; though it is inferior in strength, and slower in its effects, than strong caustic volatile alkali, yet experience has proved that it is sufficiently strong to effect a speedy and complete cure.

Oil should not be given before or during the exhibition of the hartshorn; for it would weaken its effects, or combine with it and make soap. That the volatile alkali, properly administered, will in a short time cure the bite of any snake, or the sting of a spider, or any other venomous insect, is a medical fact as well established as that the Peruvian bark will cure an intermittent fever. There are exceptions to all general rules, and probably more to the latter than to the former. With the exception of a few extreme cases in which the bite proves instantly mortal, either from the uncommon virulence of the poison, the peculiar nature of the part which it is applied, or the operation of fear, the volatile alkali may be depended on to afford a certain and speedy cure. Of this we have authentic evidence in the books referred to above, which state cures performed in the East Indies by means thereof, even in cases where the poison had advanced so far that mechanical force was necessary to unlock the jaw before the medicine could be introduced.

Such persons as have no access to these authorities, or are slow to believe the records of distant events, are requested, for their further satisfaction, to inform themselves of the particulars of the cure beforementioned, as having taken place on Charleston Neck since the commencement of June, 1803. On inquiry they will find that the most alarming symptoms were removed in a few hours by the unassisted operation of this single remedy.

That volatile alkali should always succeed is not to be expected; but in nine cases out of ten its failure, on a proper examination of every circumstance, would probably be found to arise from one or more of the following circumstances: either the medicine given as volatile alkali was spurious, or inferior in its kind, or weakened by being frequently opened, or insecurely corked; or that it had been given in too small doses, or at too long intervals. Such persons as design to give it a trial are requested to be minutely attentive to each of these particulars.

As the hydrophobia following the bite of a mad dog has resisted all the remedies hitherto used for its cure, it is submitted to physicians whether, on principles of analogy, it would not be well to try the effects of volatile alkali, rather than than resign a patient to his fate, or repeat the medicines which on frequent trials have always been found unavailing? A doubtful remedy is better than none. He who does not do all in his power to save a life, especially one committed to his care, is guilty of a species of murder.

I shall be obliged by information of the result of any experiments that may be made in consequence of this communication.

